# MRI in multiple myeloma: a pictorial review of diagnostic and post-treatment findings

**DOI:** 10.1007/s13244-016-0492-7

**Published:** 2016-05-10

**Authors:** Julie C. Dutoit, Koenraad L. Verstraete

**Affiliations:** Department of Radiology, MR -1K12, Ghent University Hospital, De Pintelaan 185, B-9000 Ghent, Belgium

**Keywords:** Multiple myeloma, Magnetic resonance imaging, Dynamic contrast-enhanced MRI, Diffusion weighted imaging, Response assessment

## Abstract

Magnetic resonance imaging (MRI) is increasingly being used in the diagnostic work-up of patients with multiple myeloma. Since 2014, MRI findings are included in the new diagnostic criteria proposed by the International Myeloma Working Group. Patients with smouldering myeloma presenting with more than one unequivocal focal lesion in the bone marrow on MRI are considered having symptomatic myeloma requiring treatment, regardless of the presence of lytic bone lesions. However, bone marrow evaluation with MRI offers more than only morphological information regarding the detection of focal lesions in patients with MM. The overall performance of MRI is enhanced by applying dynamic contrast-enhanced MRI and diffusion weighted imaging sequences, providing additional functional information on bone marrow vascularization and cellularity.

This pictorial review provides an overview of the most important imaging findings in patients with monoclonal gammopathy of undetermined significance, smouldering myeloma and multiple myeloma, by performing a ‘total’ MRI investigation with implications for the diagnosis, staging and response assessment.

*Main message*

*• Conventional MRI diagnoses multiple myeloma by assessing the infiltration pattern.*

*• Dynamic contrast-enhanced MRI diagnoses multiple myeloma by assessing vascularization and perfusion.*

*• Diffusion weighted imaging evaluates bone marrow composition and cellularity in multiple myeloma.*

*• Combined morphological and functional MRI provides optimal bone marrow assessment for staging.*

*• Combined morphological and functional MRI is of considerable value in treatment follow-up.*

## Introduction

Multiple myeloma (MM) is a plasma cell dyscrasia, characterized by a proliferation and accumulation of monoclonal plasma cells [1]. The disease evolves from an asymptomatic premalignant stage, monoclonal gammopathy of undetermined significance (MGUS), over smouldering multiple myeloma (SMM), to symptomatic MM with end-organ damage, such as hypercalcemia, renal impairment, anaemia and bone disease [2, 3].

The diagnosis of MM mainly relies on the demonstration of bone marrow plasmacytosis and/or demonstration of monoclonal proteins (M-proteins) in the serum or urine and/or detection of end-organ damage, especially (lytic) bone lesions [1], based on the International Myeloma Working Group (IMWG) diagnostic criteria reported in 2014 [4–6].

Conventional radiographs used to be the gold standard in the detection of bone lesions in myeloma. However, the detection limit and sensitivity of conventional radiography for (lytic) bone lesions is low [7]. In the past 10 years, advances have been made in imaging technology, with a more widespread use of magnetic resonance imaging (MRI), low dose multidectector computed tomography (MDCT) and ^18^F-fluoro-deoxyglucose positron emission tomography (^18^F-FDG PET)/^18^F-FDG PET-CT to assess lytic bone lesions, but also early stages of bone marrow infiltration [4].

MRI remains the most sensitive and specific imaging method for the detection of bone marrow infiltration, before mineralized bone has been destroyed [8]. The presence of more than one focal lesion on MRI (> 5 mm) is therefore enough to define MM [4, 9]. However, there is an increasing awareness that anatomical approaches based on measurements of tumour size have significant limitations for assessing therapy response [10]. There is evidence that the detection rate and overall performance of MRI could be enhanced when information on bone marrow cellularity and vascularization is added, by applying functional MRI techniques, such as diffusion weighted imaging (DWI) and dynamic contrast-enhanced imaging (DCE-MRI), respectively [11, 12].

In this pictorial review, a practical guideline for a ‘total’ MRI evaluation is presented, including information from conventional MRI, DCE-MRI and DWI, providing a complete morphological and functional evaluation of patients with plasma cell disease.

## MR imaging techniques

### Conventional SE MRI

**Fig. 1:**
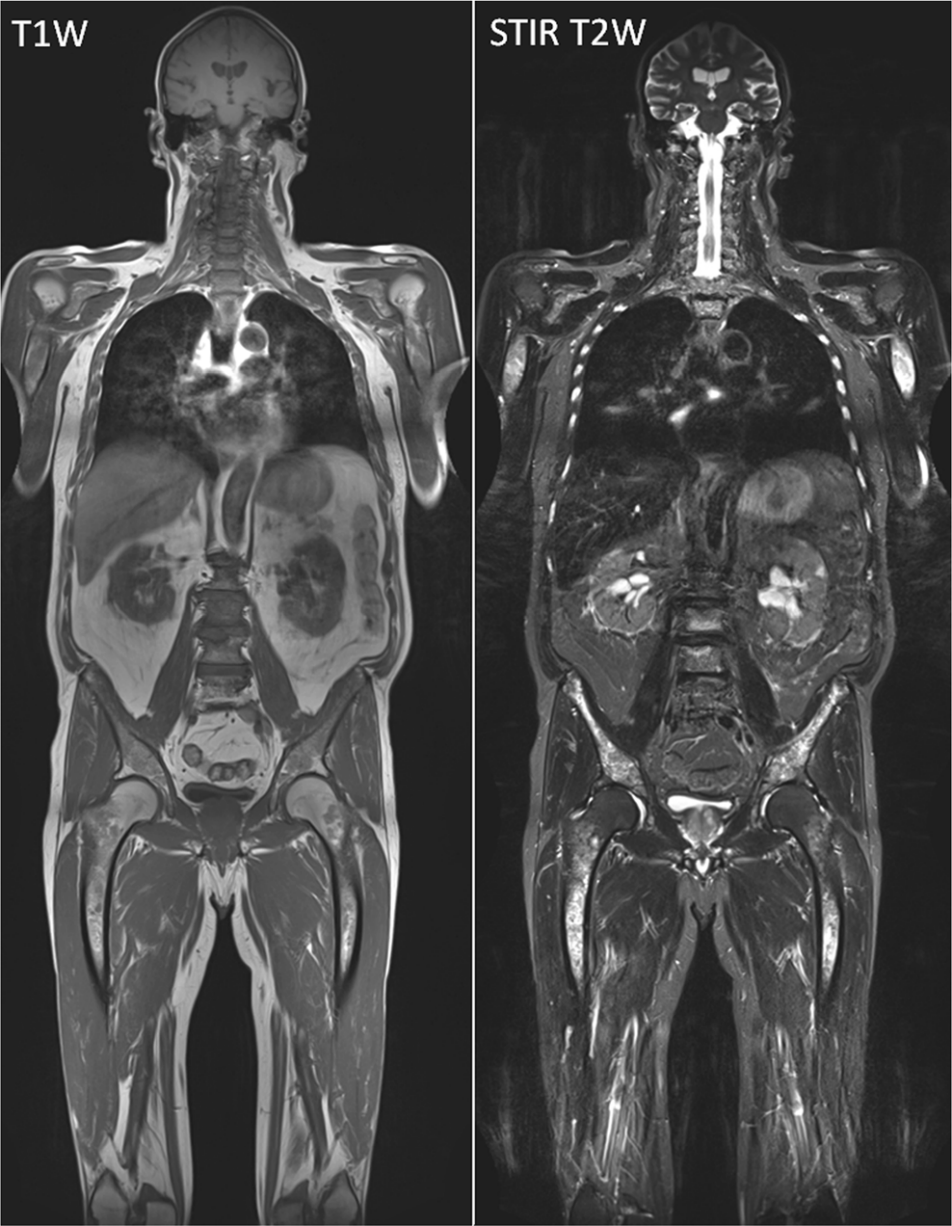
Coronal T1-weighted (*left*) and T2-weighted STIR (*right*) coronal whole body MR images displaying a diffuse marrow infiltration in the spine, pelvis, femora, humeri, ribs and scapulae. Lesions appear hypointense on T1-weighted images and hyperintense on the STIR images. Remark the good contrast resolution of STIR images in revealing infiltration of the ribs: ‘white ribs sign’

The most frequently used MR sequences for the evaluation of bone marrow are conventional T1-weighted spin-echo (T1-weighted) and T2-weighted spin-echo (T2-weighted) sequences. The signal intensities on MR images are based on the proportionate composition of red and yellow marrow and to a lesser extent mineralized matrix [13, 14] (Fig 1).

**Fig. 2:**
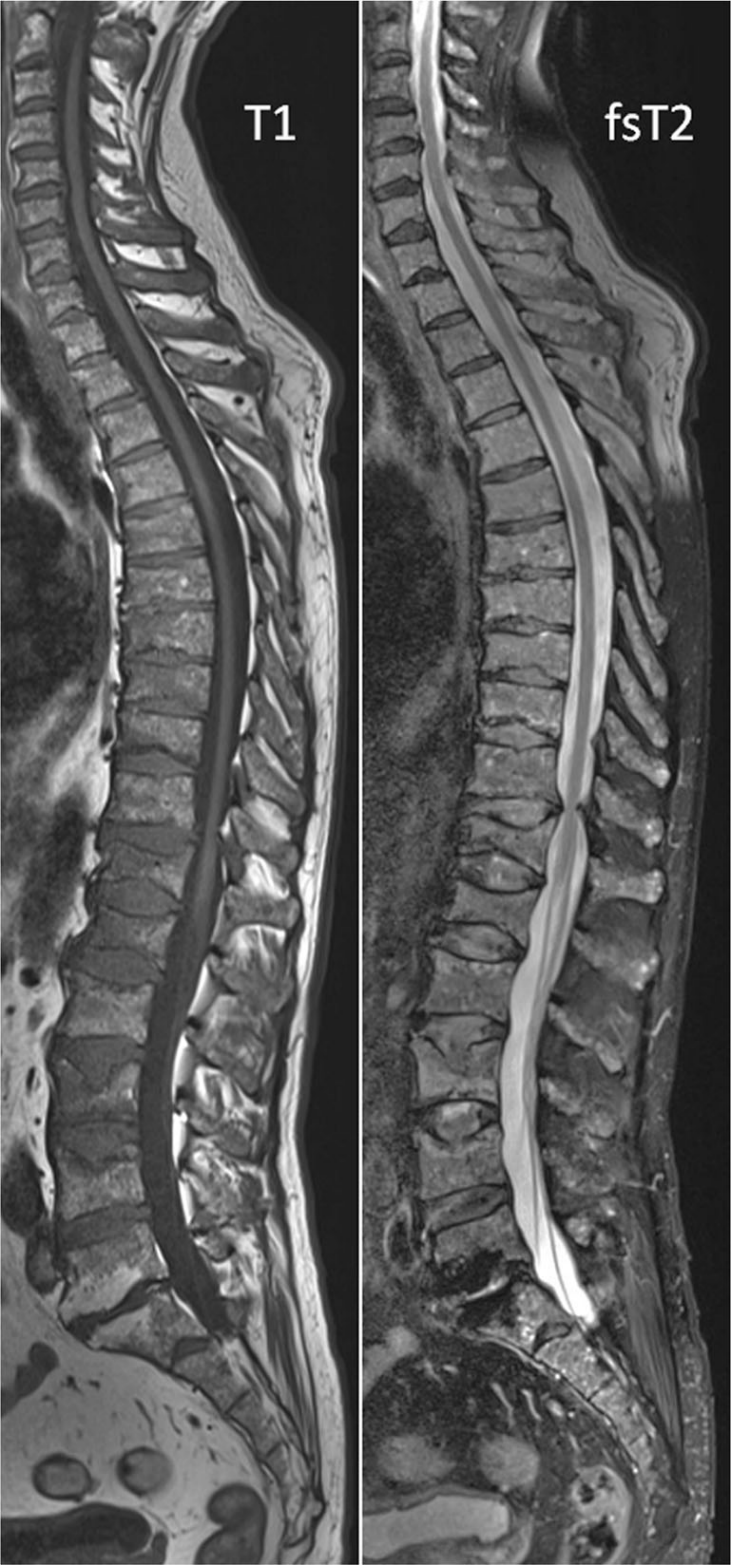
Sagittal T1-weighted (*left*) and fat-suppressed T2-weighted (*right*) images of the spine displaying a diffuse bone marrow infiltration of the cervical, thoracic, lumbar and sacral spine with low signal intensity on T1- and intermediate to high signal intensity on fat-suppressed T2-weighted images

T1-weighted images are best to evaluate bone marrow because of the high fat content interspersed with hematopoietic elements, appearing hyperintense compared to muscle and intervertebral disc [15]. Fat protons have relatively long T2-relaxation times and appear iso- to hypointense compared to the subcutaneous fat on T2-weighted images [13]. Bone marrow contrast can be accentuated by using fat-suppression (fs) sequences. The chemically selective fat-suppression technique STIR tends to produce more homogenous fat-suppression than T2-weighted images with fat suppression [15]. Lesions with a high cellularity and high amount of water are readily visible on STIR images as hyperintense structures, with corresponding hypointensity on T1-weighted images [13, 16] (Fig 2).

Our standard myeloma whole body conventional MR protocol consists of T1-weighted and STIR images of the body in the coronal plane and sagittal T1- and fsT2-weighted images of the spine (Figs. 1 and 2).

### Dynamic-contrast enhanced MRI

DCE-MRI can be used to detect and monitor changes in bone marrow microcirculation as a result of myeloma-induced angiogenesis and changes in tumour blood flow and vascular permeability. The process of angiogenesis plays a role in the development, growth, and prognosis of hematologic malignancies [17]. The newly formed blood vessels are disorganized, fragile and tortuous with an increased permeability due to large fenestrations in the vessel walls [18].

DCE-MRI is an imaging technique that investigates in a non-invasive manner the neoangiogenesis of tumoral tissue by providing clinically useful information on tissue vascularization, perfusion, capillary permeability and composition of the interstitial space [19]. A bolus of gadolinium-based contrast medium is injected intravenously, and imaging is performed during and immediately after injection by making sequential images of the spine, eight parallel sagittal slices per series followed by 74 consecutive series during the first 2 min. Typically a fat-saturated T1-weighted ultrafast sequence of the thoracolumbar spine is executed, followed by a static fat-saturated spin-echo T1-weighted sequence [19, 20].

The temporal changes that occur during passage of the contrast bolus are depicted in time-intensity curves (TIC). These curves provide useful information on the amount of contrast medium diffusing from the intravascular into the extravascular space and on the kinetics of this process. During the first pass of the contrast bolus, there is an immediate unidirectional flow from the intravascular to the extracellular space, called ‘wash-in’. After the first pass a decrease in concentration of contrast medium and signal intensity occurs by intravascular dilution, leakage into the tissues and renal clearance, resulting in a steady state. If the intravascular concentration of contrast medium drops under the interstitial concentration level, diffusion takes place in opposite direction until all contrast is eliminated: this is called ‘wash-out’ [19–21].

**Fig. 3:**
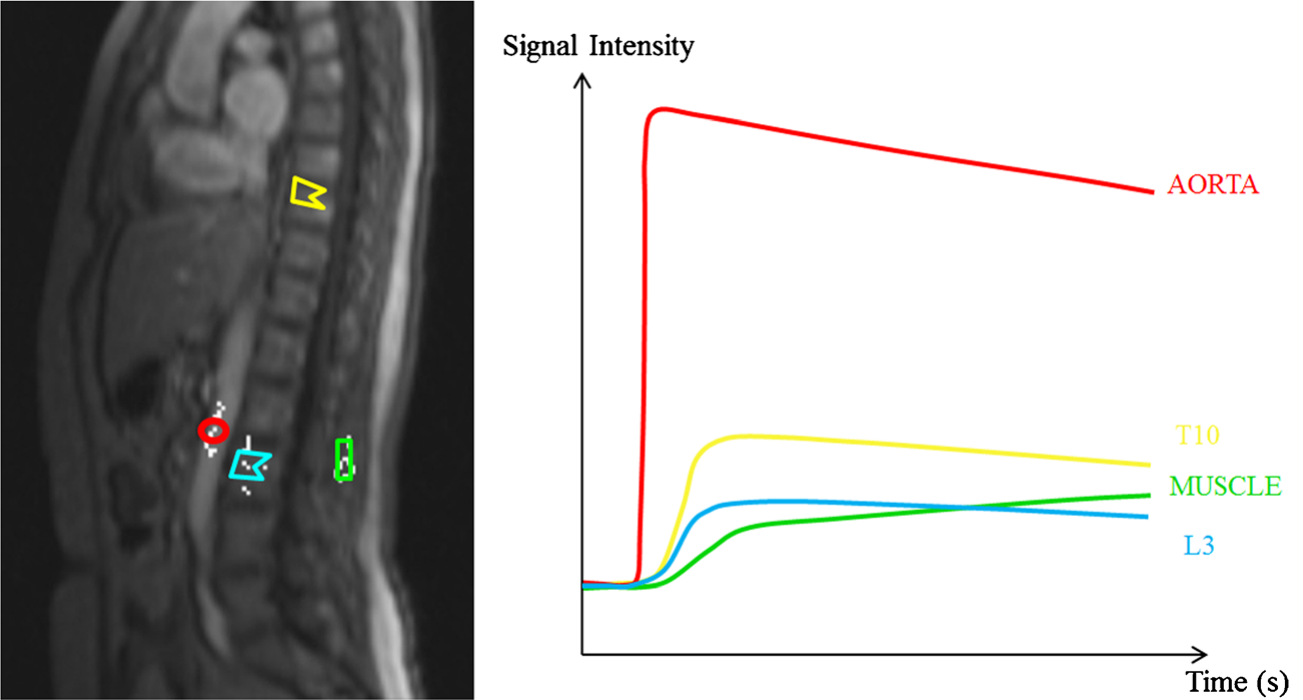
A parasagittal dynamic contrast-enhanced MR image is displayed with regions of interest (ROI) drawn in the aorta (*red circle*), paraspinal muscle (*green rectangle*), vertebra T10 (*yellow polygon*) and L3 (*blue polygon*). ROI-selection in the vertebrae should exclude the entrance of the vertebral vessels and the endplates. This method provides a corresponding time-intensity curve displayed on the right, providing information on tissue vascularization, perfusion, vessel permeability and volume of the interstitial space

TICs, provided by the region-of interest method, allow calculation of semi-quantitative parameters that depict the characteristics on vascularity and perfusion of myeloma infiltrated bone marrow (Fig 3) [19, 20, 22]. A quantitative analysis of DCE imaging data can be performed using mathematical models that take into account the enhancement kinetics of the artery supplying the area studied. Pharmacokinetic models depict changes in tissue contrast concentrations instead of signal intensity changes over time [23, 24].

**Fig. 4:**
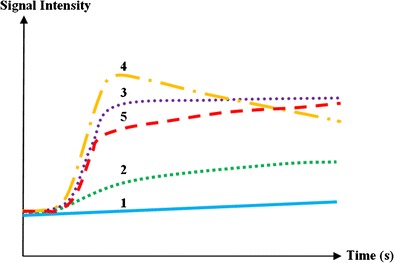
Five types of time-intensity curves (TICs) have been described. Type 1 (*blue*) demonstrates no enhancement, whereas type 2 (*green*) illustrates a slow sustained enhancement. Type 3 (*purple*), 4 (*orange*) and 5 (*red*) are characterized by a steep and fast first pass enhancement; in type 3 this is followed by a sustained late enhancement; in type 4 this is followed by a wash-out of contrast medium caused by a small interstitial space. The steep wash-in of type 5 is followed by a stable late enhancement

This review is focusing on the qualitative interpretation of TICs based on the shape of the curve, providing valuable information on the degree of contrast diffusion. A classification of five types of curves is described in the literature (Fig 4) [20, 22]. MM bone marrow infiltration is typically characterized by type 4 curves, and less frequently type 3 or 5 curves. Type 1 and 2 curves typically occur in healthy persons or patients with MGUS [19, 20, 22, 25].

### Diffusion weighted imaging

DWI is a technique derived from MRI and is increasingly being used to assess bone marrow because of its sensitivity to cell density, the relative content of fat and marrow cells, water content and bone marrow perfusion [26]. The signal intensity of DWI relies on the stochastic Brownian motion or self-diffusion of water molecules at microscopic level within tissues [27]. This Brownian motion of water molecules in the interstitium allows quantifying the cellularity of tissues: cellular tissues like solid tumours are characterized by a small interstitium, which restricts water motion [10, 28].

Sagittal diffusion weighted images of the spine in this review are obtained with echoplanar imaging using different b values (0-200-400-600-1000 s/mm^2^). The signal losses in the tissues are proportional to both free motion of water molecules and the diffusion gradient strength used [27].

**Fig. 5:**
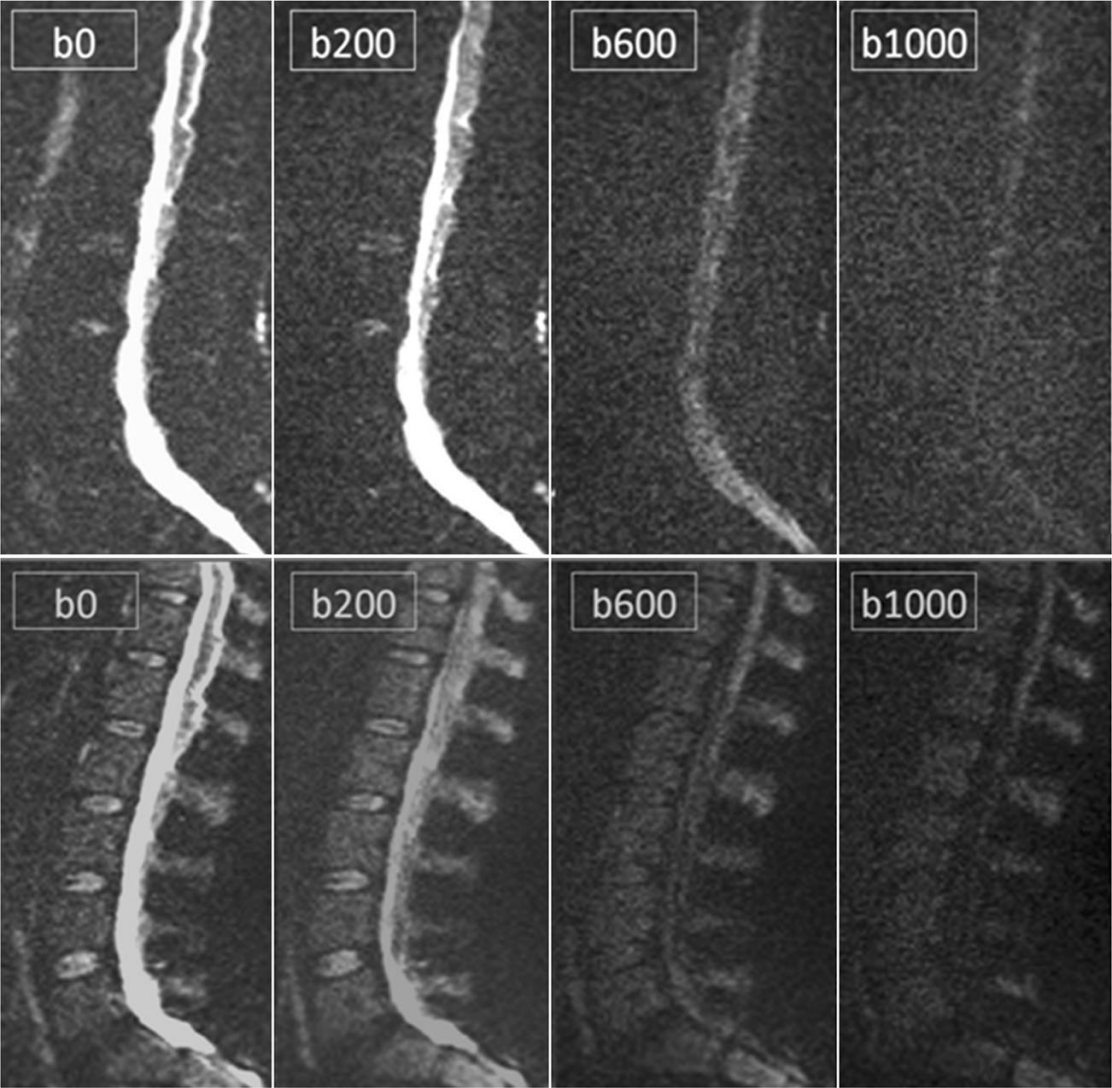
Example of diffusion weighted b-value images b0-b200-b600-b1000. The upper sequence demonstrates the b-value images of a patient with monoclonal gammopathy of undetermined significance, the lower sequence images belong to a patients with multiple myeloma. The signal intensity of myeloma lesions are typically high on b-value images, due to the low amount of fat cells, increased cellularity and water amount. Remark the good visibility of the vertebral bodies and spinous processes in this patient with multiple myeloma

Initial assessment of bone marrow disease on DWI are usually made by visually assessing the signal intensity on high b-value images (usually b1000), as high signals correspond to bone marrow infiltration (Fig 5). A DWI investigation is scored ‘positive’ if hyperintense focal lesions or diffuse hyperintense vertebral bodies can be seen on high b-value images, indicating diffusion restriction of water in highly cellular tissue with a small interstitial space [16, 26]. This method of assessment is practically useful and clinically appealing for both radiologists and referring physicians [29], because lesions are more conspicuous on this type of images.

The quantified parameter derived from DWI is the apparent diffusion coefficient (ADC), which is a direct indicator of water motion within extracellular and intracellular space and is thus directly related to tissue cell density [30].

**Fig. 6:**
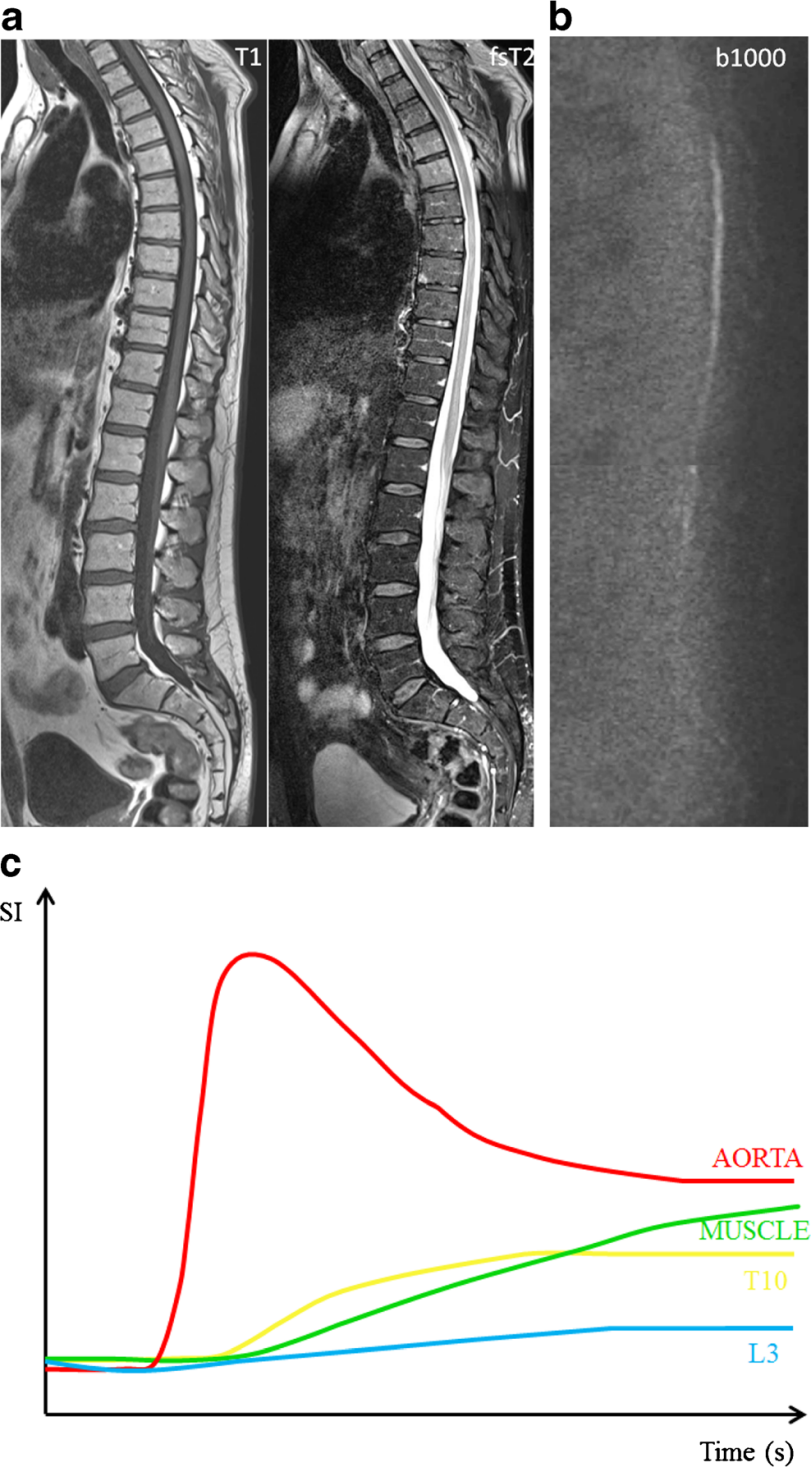
MR images of a patient with monoclonal gammopathy of undetermined significance, with 5 % plasma cells on bone marrow biopsy. **a** T1-weighted and fat-suppressed T2-weighted images of the thoracolumbar spine displaying normal bone marrow signal intensities, hyperintense on T1 and hypointense on fat-suppressed T2-weighted images. **b** DWI b1000 images with normal appearing bone marrow, low signal intensity due to the high amount of fat and low cellularity with low water diffusivity. **c** Time-intensity curve derived from DCE-MR imaging with a TIC type 1 (L3 - *blue*) and type 2 (T10 - *yellow*) curve, corresponding to a normal vascularization of the bone marrow, no signs of neoangiogenesis

Yellow marrow appears hypo-intense on b-value images with low ADC values, related to low cell density, with an abundance of fat cells, and reduced water proton diffusivity, due to the hydrophobic nature of fat and lower bone marrow perfusion compared to red marrow. Red bone marrow has a lower adiposity and higher water content, which contributes to the higher signal intensity on b-value images and higher ADC values [16, 26] (Fig 6).

## Monoclonal gammopathy of undetermined significance and smouldering myeloma

**Fig. 7:**
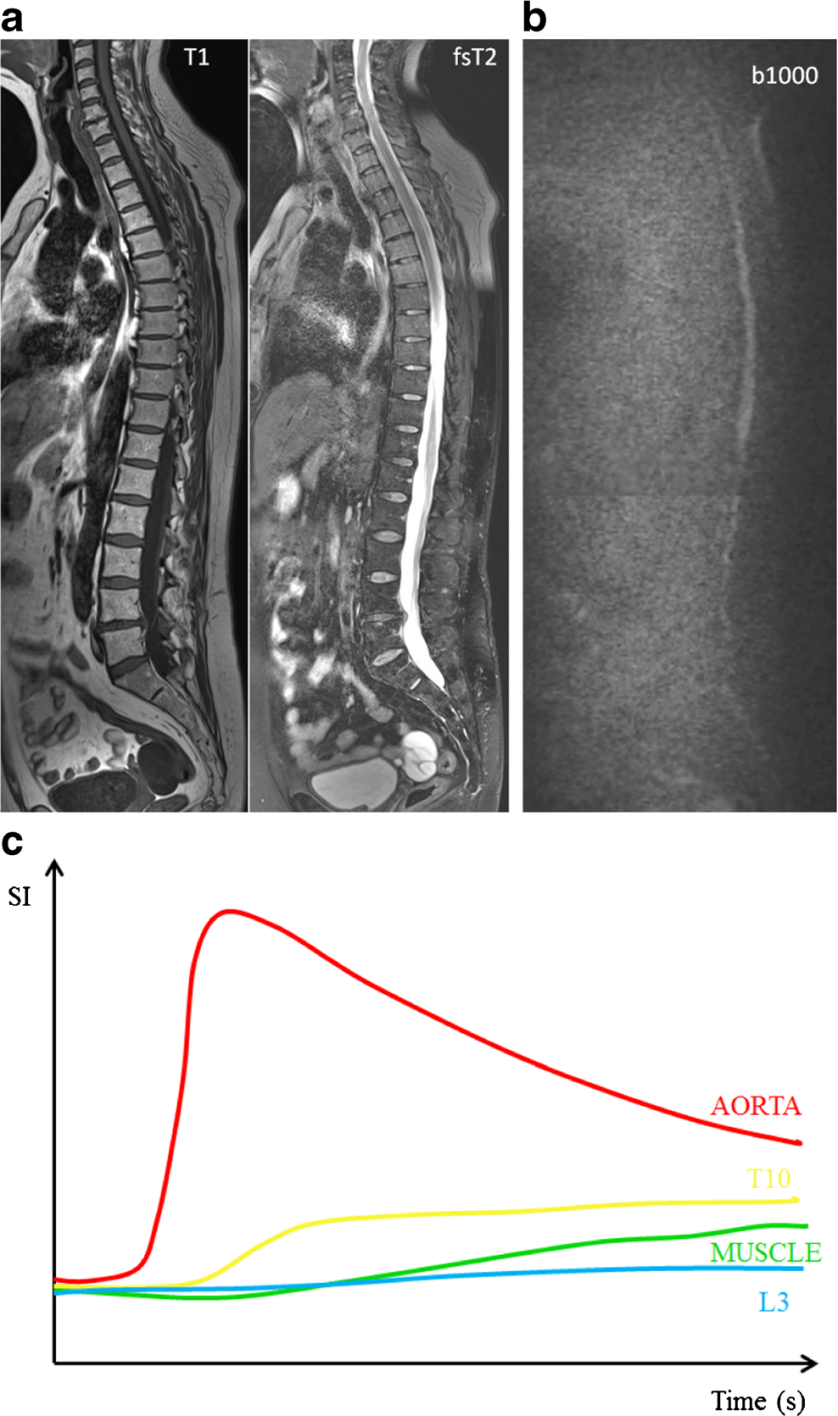
MR images of a patient with smouldering multiple myeloma, 15 % plasma cells on bone marrow biopsy. **a** T1-weighted and fat-suppressed T2-weighted images of the thoracolumbar spine displaying altered bone marrow signal intensities, with slight diffuse signal decrease on T1 images without significant changes on T2-weighted images. **b** Normal signal intensity on b1000 images, due to the residual high amount of fat cells in the bone marrow. **c** Time-intensity curve derived from DCE-MR imaging with TIC type 1, corresponding to a normal vascularization of the bone marrow without signs of neoangiogenesis

Patients with MGUS have a normal bone marrow appearance on MRI, hyperintense on T1-weighted images (high fat content) and hypointense on fsT2-weighted images (low water content) [9, 16, 17] (Figs 6a and 7a). The presence of diffuse infiltration of the bone marrow, and especially the presence and number of focal lesions are significant prognostic factors for progression from MGUS or SMM to MM [9, 11, 31]. The IMWG consensus statement now recommends that SMM patients with more than one unequivocal focal lesion (diameter > 5 mm) should be considered to have symptomatic myeloma that requires treatment. Patients with equivocal focal lesions should repeat the MRI after 3–6 months and in case of MRI progression, patients should be considered as symptomatic patients who need therapy. Patients with MGUS and MRI-identified focal lesions seem to have an increased risk of progression to myeloma. To date, the IMWG has not yet recommended MRI as part of the routine workup for patients with MGUS unless there are clinical features that increase suspicion of progression to MM [7].

There is no increased bone marrow perfusion in patients with MGUS compared to healthy controls [32]. Patients with MGUS typically provide type 1 and type 2 TICs, as does normal fatty bone marrow. These types of TIC represent a low and slow wash-in of contrast medium due to low vascularisation, perfusion and capillary permeability. This is followed by a plateau phase because of the slow wash-out of contrast medium from the interstitial space back in the intravascular space [20] (Figs 6c and 7c).

MGUS and SMM patients with increased microcirculation patterns on DCE-MRI appear to have a significantly higher bone marrow plasmacytosis compared to patients with a low microcirculation pattern [17]. An increased bone marrow perfusion is found in transition from SMM to symptomatic MM [32].

Patients with MGUS present with low ADC values and low signal intensity on high b-value images, due to the high restriction of water diffusion, which can be explained by a higher bone marrow fat content and lower water content. No differences could be found between patients with MGUS and SMM. Bone marrow infiltration has to be high enough to result in a decrease in fat cells, detectable on conventional and diffusion-weighted MRI [16] (Figs. 6b and 7b).

## Multiple myeloma

**Fig. 8:**
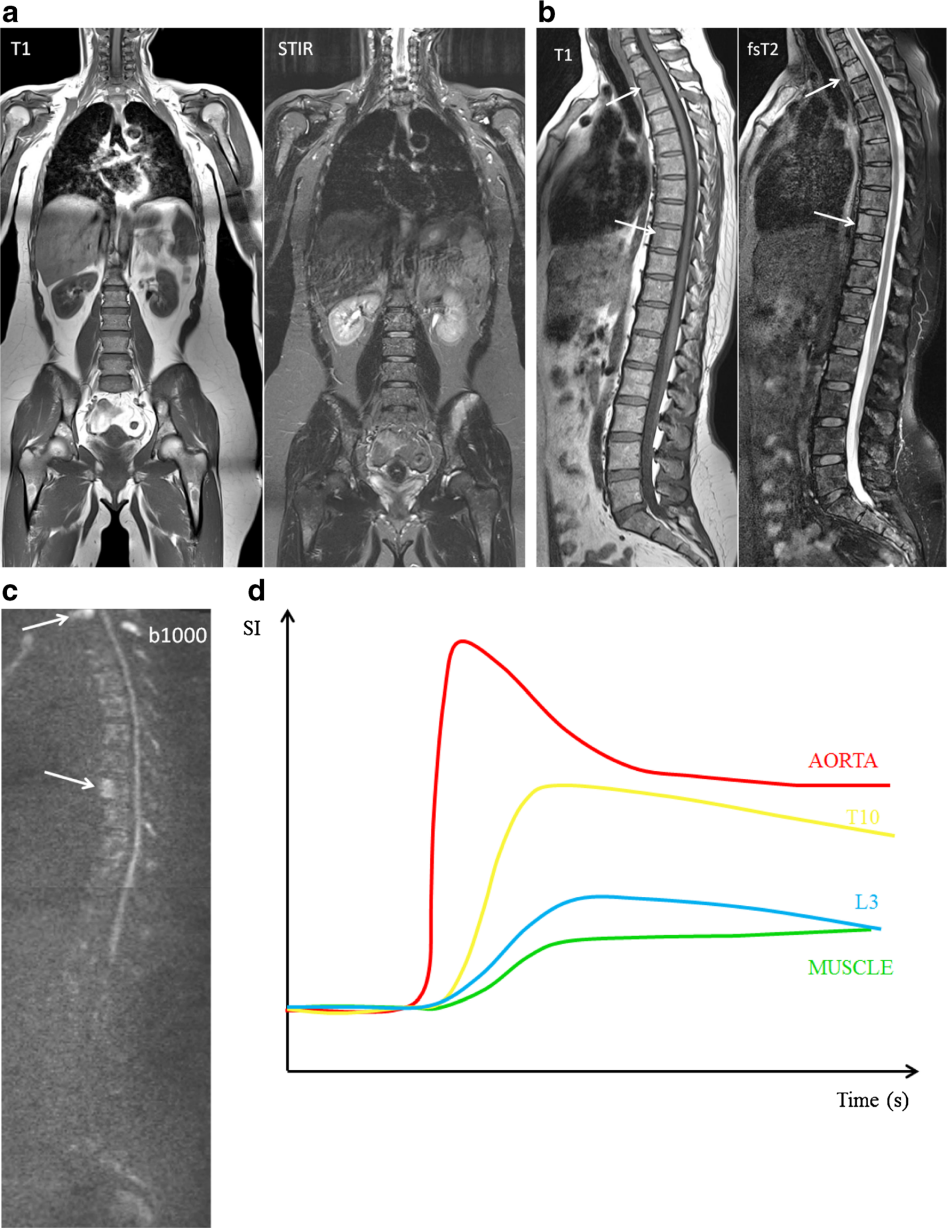
MR images of a patient with symptomatic multiple myeloma, 30 % plasma cells on bone marrow biopsy. **a** T1-weighted and T2-weighted STIR coronal images of the body and **b** T1-weighted and fat-suppressed T2-weighted images of the thoracolumbar spine displaying a significant decrease of signal intensity on T1-weighted images with a corresponding increased signal intensity on T2-weighted images, diffuse infiltration pattern, and also remark the additional focal lesions in the vertebral bodies of T2 and T9 (*arrows*). **c** Increased signal intensity on DWI b1000 images, due to a decrease in bone marrow fat cells, increased cellularity, and water amount with corresponding increase in water diffusivity. **d** Increased angiogenesis, perfusion and vascular permeability displayed in a time-intensity curve type 4, with early wash-out of the contrast medium

Typical myeloma lesions appear hypointense on T1-weighted images, due to the lower fat content, typically lower than the muscle and intervertebral disc. On fat-suppressed T2-weighted images, the lesions appear rather hyperintense due to the high cellularity and high amount of water [13, 16] (Fig 8a, b). Predilection sites of MM are the axial skeleton, spine and pelvis, but also the extra-axial skeleton, ribs, shoulder, skull and proximal femora. This explains the whole body approach for an adequate assessment of extent of disease [20, 33]. The infiltration of the ribs is best appreciated on T2-weighted images with fat suppression, appearing bright: ‘white ribs sign’ (Figs. 1 and 8a).

**Fig. 9:**
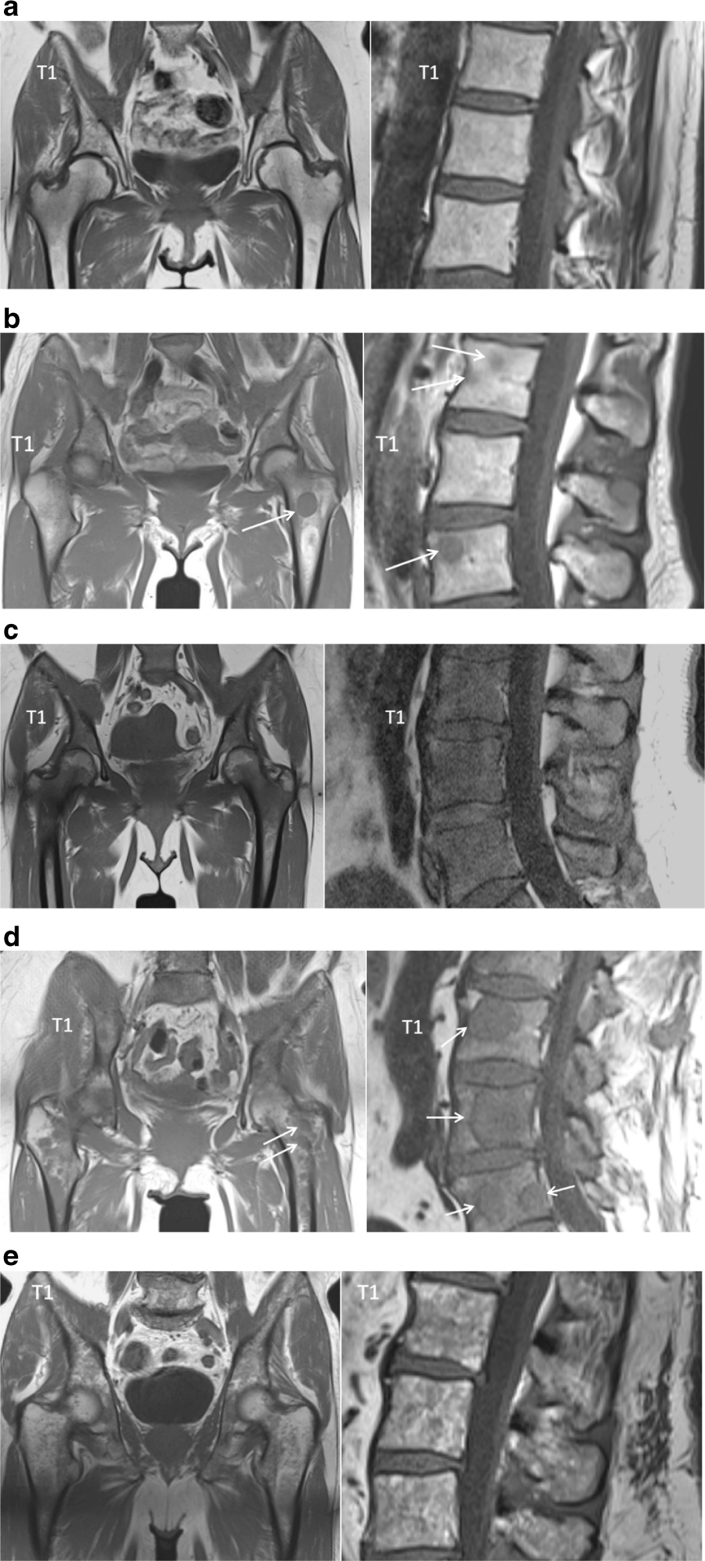
Bone marrow infiltration patterns. **a** Normal appearing bone marrow, **b** focal (*arrow*) and **c** diffuse myeloma infiltration pattern, **d** combination of focal and diffuse infiltration and finally **e** salt-and-pepper infiltration pattern

Five different patterns of bone marrow infiltration in multiple myeloma have been identified on MR imaging, including a normal appearing marrow, focal infiltration, diffuse disease, salt-and-pepper involvement or combined focal and diffuse infiltration (Fig 9) [34, 35]. These infiltration patterns on MR images have been found to correlate to histological findings on bone marrow biopsies [35, 36]. In 28 % of the multiple myeloma patients, a normal appearing bone marrow signal is found in all sequences with high signal intensity on T1-weighted and low signal on T2-weighted sequences with fat suppression (Fig 9a). Focal lesions are areas of high signal intensity on fsT2-weighted sequences. These correspond to areas of low signal intensity on unenhanced T1-weighted images. In a few cases, isointense signal is found on T1-weighted images (Fig 9b). The diffuse infiltration pattern is characterized by a homogenous decrease of signal on T1-weighted images and increased signal intensity on T2-weighted images with fat suppression (Fig 9c). In cases of high-grade diffuse involvement, the signal intensity is nearly equal to or lower than the signal intensity of the intervertebral disc on T1-weighted images due to the increase of water and decrease of fatty components. A focal and diffuse infiltration pattern can be found in about 11 % of the patients. On T1-weighted images, the bone marrow signal intensity is diffusely decreased with additional foci interspersed. Those foci are often better demarcated on fat-saturated images (Fig 9d). In about 3 % of the cases on T1-weighted images but also T2-weighted sequences, the bone marrow presents with an inhomogeneous salt-and-pepper pattern (Fig 9e) [35].

MM was the first hematological malignancy in which the diagnostic and prognostic relevance of angiogenesis was demonstrated [37]. Neoangiogenesis in multiple myeloma is responsible for the increase in bone marrow perfusion, reflected by increasing DCE-MRI related (semi-) quantitative parameters [38–41]. The most typical TIC in patients with MM is type 4 (Fig 8d). This curve represents a steep wash-in of contrast medium, due to the high vascularization and perfusion with leakage through the highly permeable capillaries, followed by an early wash-out back into the intravascular space because of the small interstitial space with closely packed plasma cells. Type 3 and type 5 occur in myeloma patients with a residual moderate size of the interstitial space, characterized by a wash-out plateau-phase or increasing enhancement [19, 20].

**Fig. 10:**
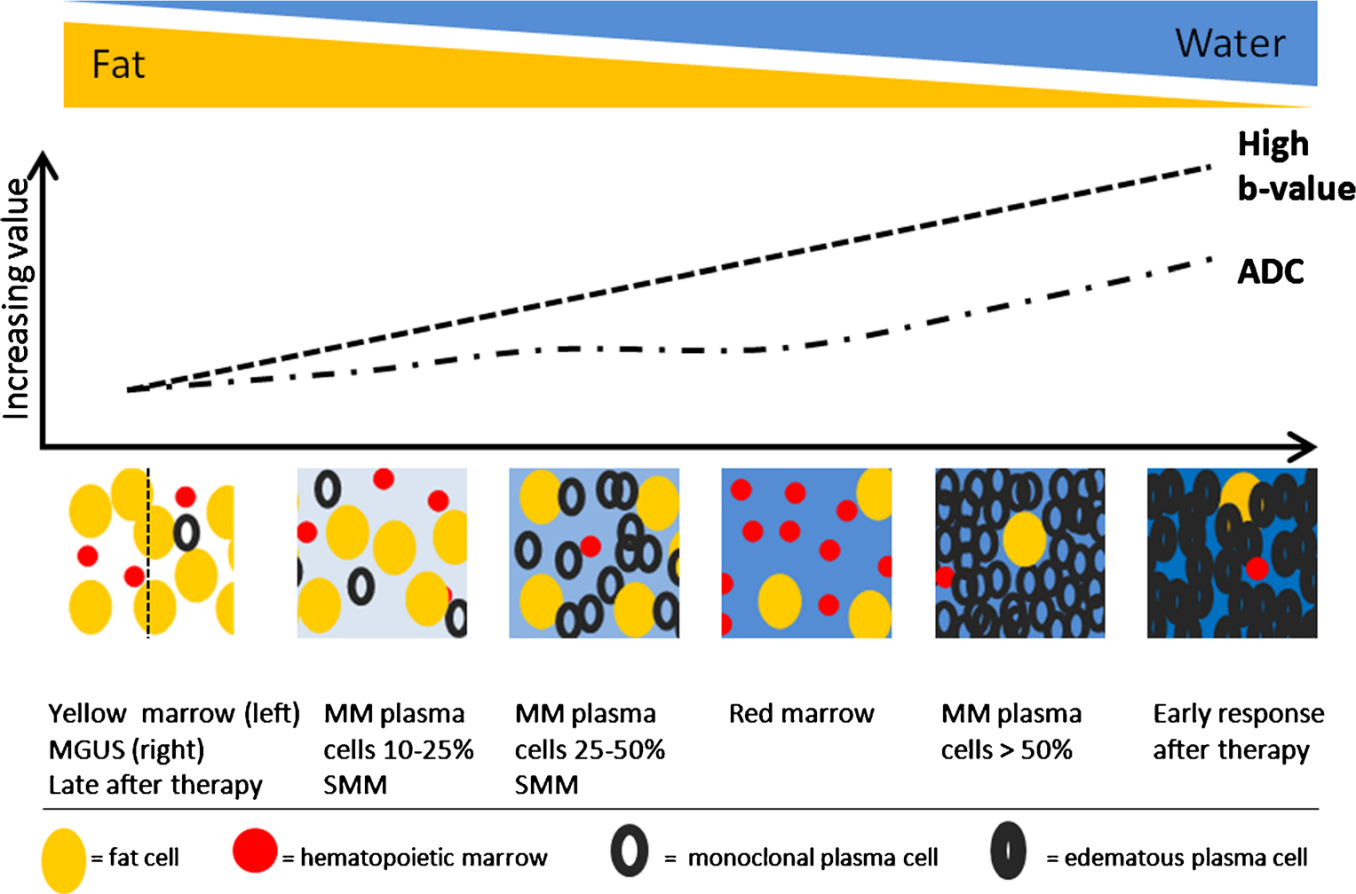
This figure displays the change in signal intensity on b images and ADC value during the disease course from monoclonal gammopathy of undetermined significance, over smouldering to myeloma-infiltrated bone marrow with plasma cell percentages of 10-25 %, 25-50 % and >50 %, followed by the changes early and late after therapy, and compared to normal red and yellow marrow. The different diffusion characteristics are explained by changes in the amount of fat and interstitial water, with an increasing water proton diffusivity as fat decreases and interstitial water increases

Myeloma infiltration of the bone marrow is characterized by the highest signal intensities on b-value images and highest ADC values compared to normal bone marrow. This is likely to be related to the absence of fat cells, higher proton density, destruction of trabecular bone, and high cellularity. The lesions appear as areas of increased diffusivity compared to the very low diffusion of normal background marrow (Fig 8c) [16, 26]. The significant decrease in signal intensity on T1-weighted images occurs rather late in the evolution, in patients with a plasma cell percentage of 50 % or more, correlating with a high signal intensity on fsT2-weighted and high b-value images with high ADC values. The bone marrow infiltration has to be high enough to result in a decrease in fat cells, detectable on conventional and diffusion weighted MRI (Fig 10) [16].

## Response assessment

**Fig. 11:**
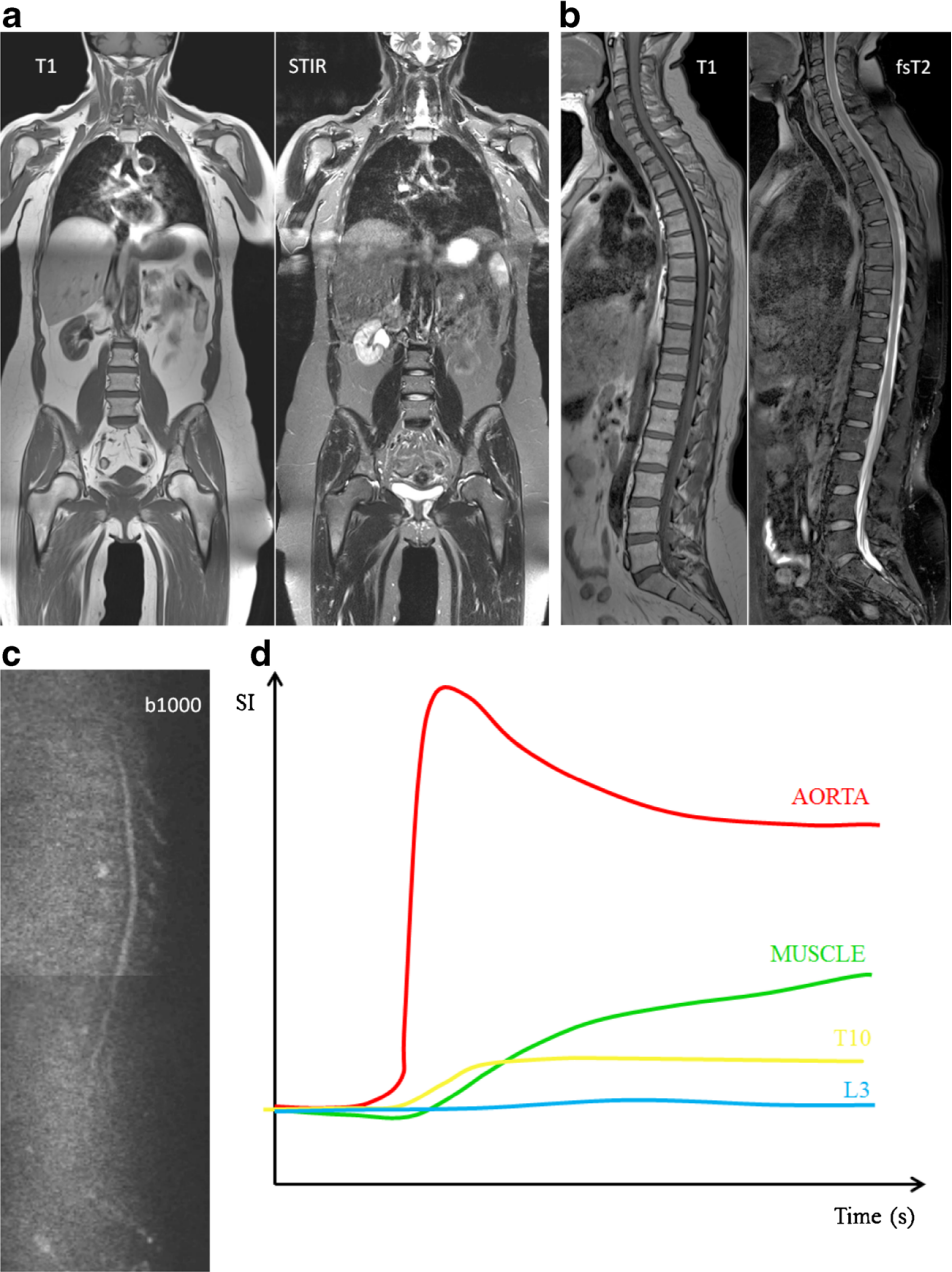
These images present the same patient as in Fig. 8, with response to therapy, 100 days after autologous stem cell transplantation, with 5 % plasma cells on bone marrow on biopsy. **a** T1-weighted and T2-weighted STIR coronal images of the body and **b** T1-weighted and fat-suppressed T2-weighted images of the thoracolumbar spine, demonstrating a clear increase in signal intensity on T1-weighted and decrease on T2-weighted images, due to reappearance of normal bone marrow. **c** There is a residual high signal intensity on the b1000 images, due to residual oedema and necrosis after therapy despite the return of fat cells, accompanied by **d** a decrease in perfusion due to destruction of the neovasculature with the reappearance of a type 1 (L3 - blue) and type 2 (T10 - yellow) curve

**Fig. 12:**
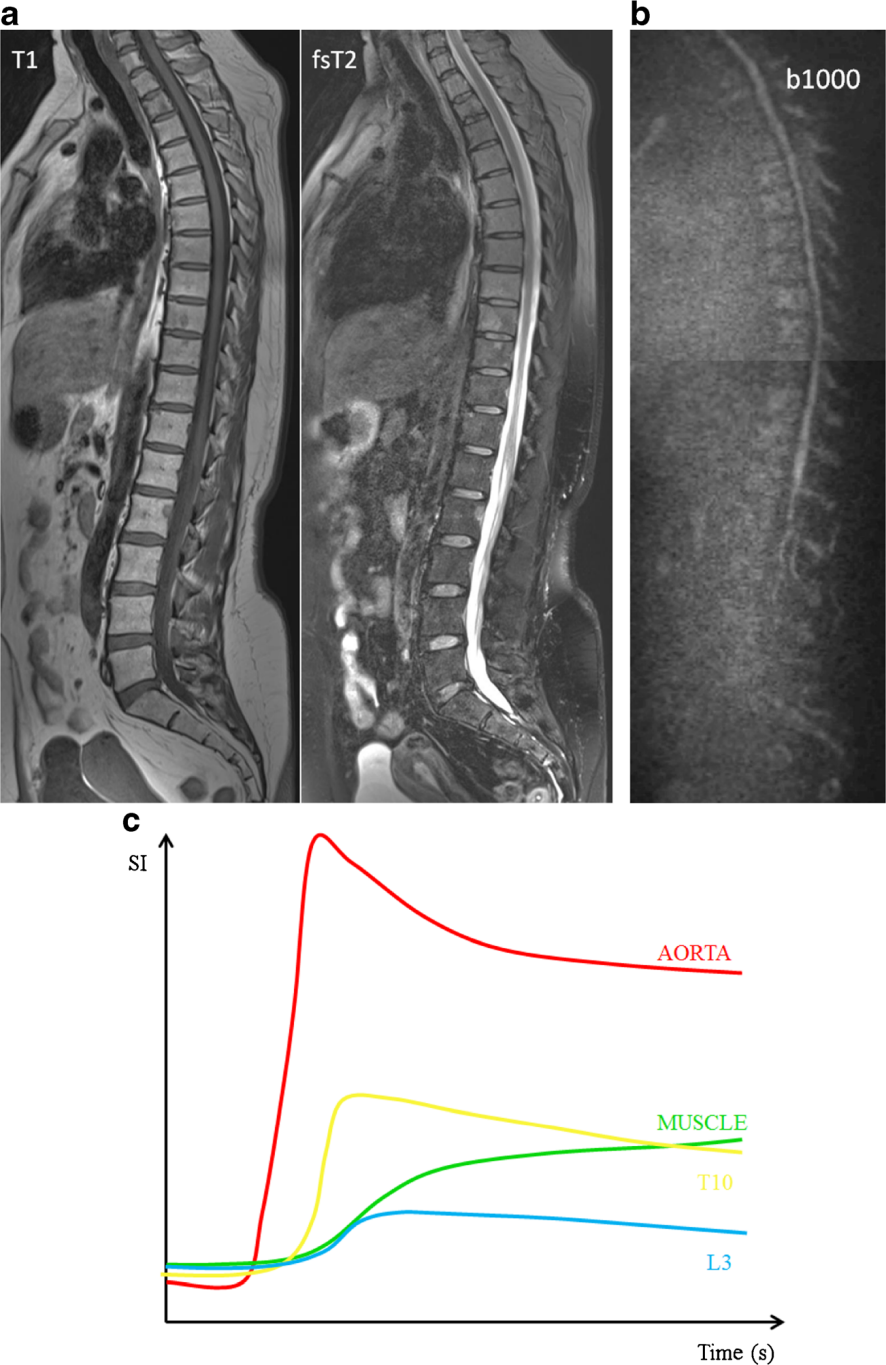
These images illustrate the same patient as in Figs. 8 and 10, with a relapse 1 year after autologous stem cell transplantation with 30 % plasma cells in the bone marrow on biopsy. **a** T1-weighted and fat-suppressed T2-weighted images of the thoracolumbar spine, with a subtle decrease in signal intensity on T1-weighted and increase on T2-weighted images, reappearance of normal bone marrow. **b** The changes are more clearly illustrated on the b1000 images with apparent increase in signal intensity, reflecting the increased cellularity and water amount compared to the previous images. **c** The changes in bone marrow composition are accompanied by the return of an active TIC type 4, due to neoangiogenesis

The follow-up of patients with neoplastic bone involvement after treatment may show evolution in infiltration patterns. Some of these changes are with no ambiguity indicative of either disease progression or response to treatment. Evolution from a normal bone marrow appearance to a focal or diffuse infiltration pattern, increase in the number and/or size of lesions and evolution from focal to diffuse neoplastic infiltration are indicative of progressive disease. Disappearance of focal lesions, decrease in their size and/or number and return from focal or diffuse patterns to a normal bone marrow appearance are indicative of response. The complete disappearance of focal lesions and diffuse infiltration may indicate complete ‘imaging’ remission, which does not necessarily correlate with complete remission at the microscopic level (Fig 11a, b). Stability in size and appearance of the marrow abnormalities after treatment should be interpreted cautiously: residual lesions may represent responsive, controlled but still active disease, or on the contrary ‘cured’ disease with persistence of ‘scar’ tissue, whether contrast material injection, study of perfusion or diffusion parameters will help in this difficult differential diagnosis remains uncertain. Relapse is characterized by the reappearance of one or several new lesions in a bone marrow that had shown a previous return to normal after therapy; sometimes it may also take the appearance of progression of a lesion that had previously shown size regression or had been stabilized under treatment (Fig 12a) [42].

**Fig. 13:**
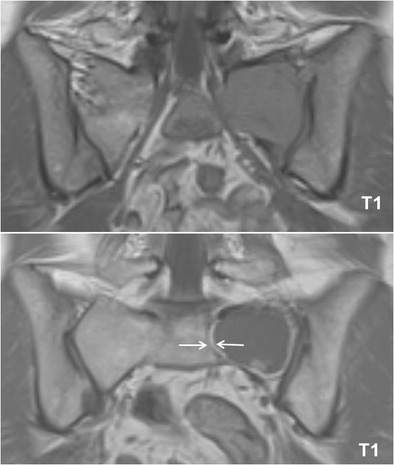
T1-weighted image of the sacrum before (*top*) and after (*bottom*) radiation therapy, presenting the appearance of a peripheral halo of fatty marrow with a characteristic high signal intensity on T1-weighted images (*arrows*), indicating response to therapy

**Fig. 14:**
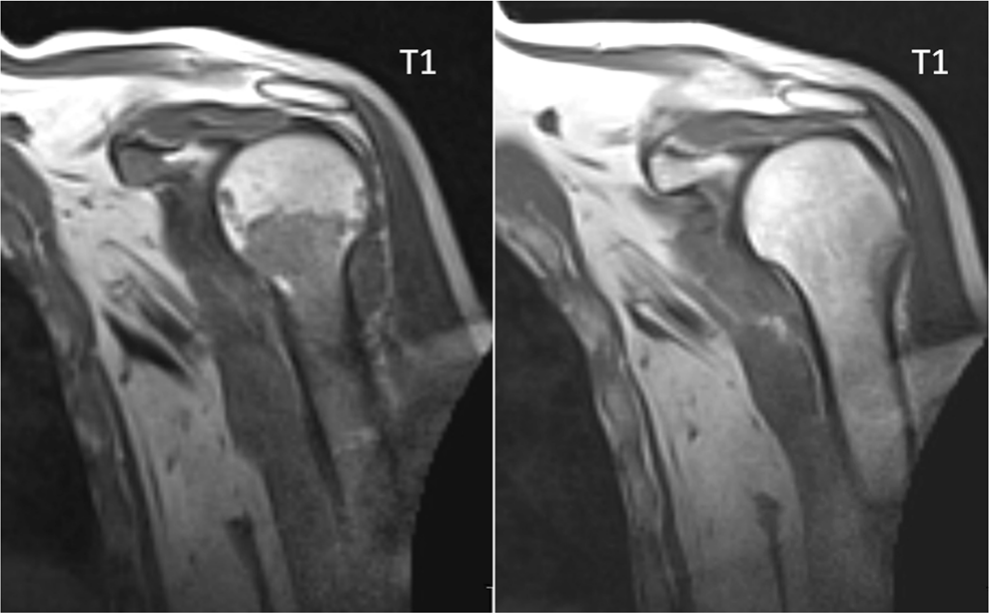
T1-weighted image of the humerus before (*left*) and after (*right*) systemic therapy. Remark the progressive fading of the bone marrow signal intensity, from hypo-intense to hyperintense signal intensity on T1-weighted imaging, indicating response to therapy

The response of focal lesions to therapy can be characterized, along with size reduction, by the appearance of a peripheral halo of fatty marrow, with characteristic high signal intensity on T1-images (Fig 13). This fatty halo sign indicates lesion response and parallels the appearance of fat within ‘response’ or ‘healing’ non-neoplastic conditions, such as chronic benign vertebral fractures, spondylodiscitis or degenerative disc disease. Lesion response sometimes does not take the appearance of shrinking, but rather progressive fading of the marrow abnormalities and return to normal marrow signal intensity within the lesion (Fig 14) [42].

**Fig. 15:**
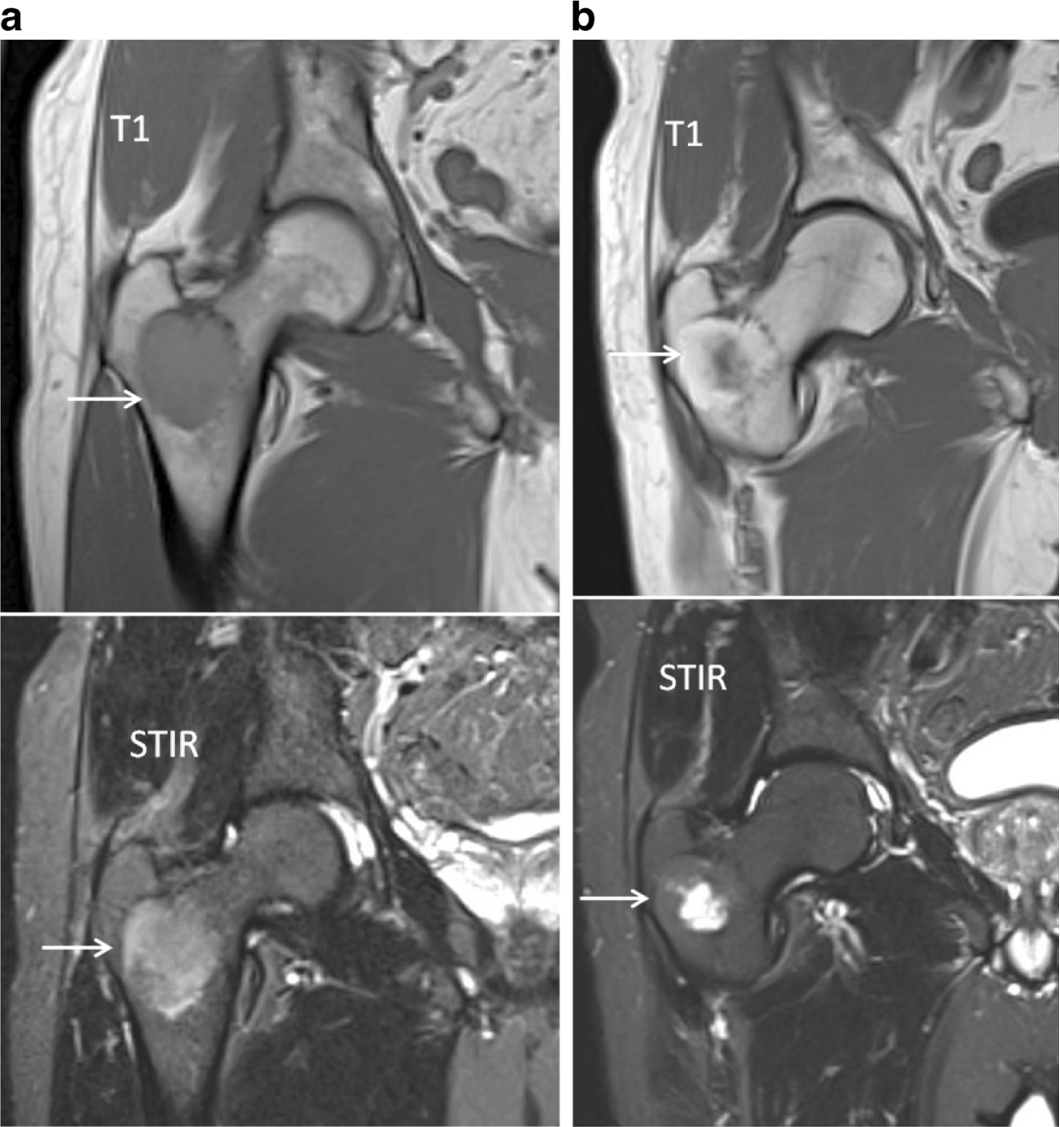
T1-weighted (*top*) and T2-weighted STIR (*bottom*) images of newly diagnosed myeloma patient **a** with diffuse infiltration of the bone marrow and a large focal lesion in the right intertrochanteric region (*arrows*). Five years after receiving systemic therapy and local irradiation on the right intertrochanteric region **b** there is an increase in signal intensity in the centre of the focal lesion on STIR images compatible with necrosis. Also remark the broad fatty halo surrounding the lesion. Both of these imaging findings indicate good response to therapy

**Fig. 16:**
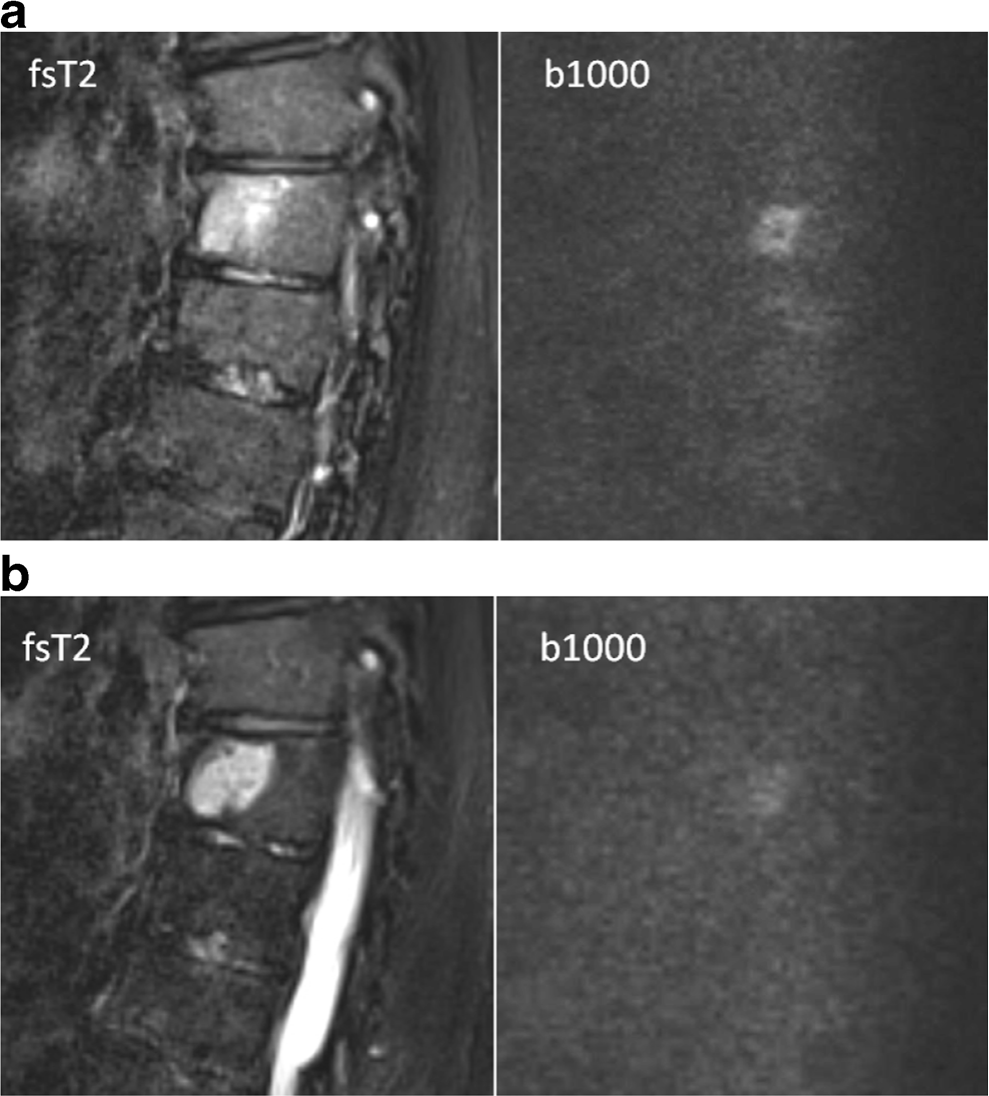
T2-weighted (*left*) images of a hyperintense focal lesion (*white arrow*) **a** in a thoracic vertebra of a newly diagnosed myeloma patient with the corresponding b1000 image (*right*), showing the focal lesion together with a slightly diffusely increased signal intensity in the all vertebral bodies. **b** After systemic treatment and local irradiation on the focal lesion the signal intensity of the focal lesion increases on the T2-weighted image, indicating response and tumour necrosis, with residual hyperintensity on the b1000 image (*right*), due to the large water amount after necrosis: T2-shine-through effect

The first days after therapy (chemotherapy or radiotherapy), the marrow undergoes cellular death and vascular congestion resulting in oedema, haemorrhage and necrosis in the bone necrosis, which appears hypointense on T1 and hyperintense on the fluid sensitive (STIR/T2-weighted) images (Fig 15) [34, 43, 44]. This is accompanied by an increase in ADC and high SI on high b-value images (T2-shine-through effects of therapy) (Fig 16) [29, 30, 45, 46]. These changes are followed by a period of fatty marrow conversion, reducing the overall increase in ADC and signal intensity on b-value images (Fig 11c) [16, 47]. Several weeks into treatment, the signal intensity on T1-weighted images of bone marrow drops again as red marrow appears on the central skeleton during the phase of hematopoietic recovery [44]. Relapse is characterized by an increase in ADC and SI on b1000 images (Fig 12b) [16].

There is a significant decrease in (semi-) quantitative parameters and thus vascularization of the bone marrow after effective therapy [20, 48, 49]. Patients with a complete response (CR) after therapy, typically present with type 1 and 2, indicating low and slow enhancing areas in the bone marrow with normal vascularization (Fig 11d). The change in type of curve is dependent on the type of treatment, depth of response to treatment, baseline neo-angiogenesis and amount of normal red marrow [20]. Relapse is characterized by the re-appearance of a type 3–5 curve, depicting active disease (Fig 12c) [20, 50].

## Summary

The value of a ‘total’ MRI investigation is clarified in Table 1.

**Table 1:**
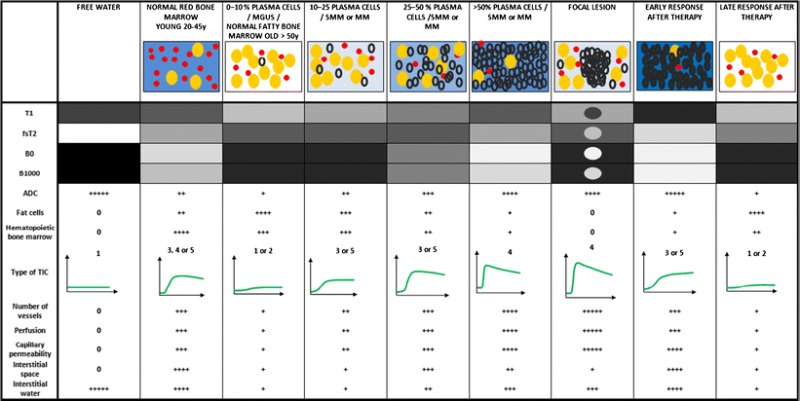
This table provides an overview of the bone marrow changes in the disease course of MM, based on information obtained from conventional MRI (signal intensity on T1- and fat-suppressed T2-weighted images), dynamic contrast-enhanced MRI (time-intensity curves and semi-quantitative parameters) and diffusion weighted images (apparent diffusion coefficient (ADC), b0 and b1000 images). The different imaging characteristics of normal bone marrow, myeloma infiltrated bone marrow with plasma cell percentage of 10–25 %, 25–50 % and >50 %, a focal myeloma lesion, are presented as signal intensities in grey-scale, followed by the changes early and late after therapy. The changes in amount of fat cells, water content, ADC-values, hematopoietic cells, interstitial space and vascularization are described in a semi-quantitative manner (0/+). This overview, is largely supported by the literature, but also partly based on experience and some explanations are currently not statistically proven

monoclonal gammopathy of undetermined significance (MGUS), T1-weighted images (T1), Fat-saturated T2-weighted images (fsT2), apparent diffusion coefficient (ADC); time-intensity curve (TIC)

Figure legend: fat cells (yellow circles), hematopoietic marrow (red dots), interstitial water (blue background), monoclonal plasma cells (black circles)

Normal bone marrow (mean age of 40 years) is characterized by a low signal intensity on T1-weighted images and a high signal intensity on fsT2-weighted and b-value images, with corresponding ADC-value of 2.94 × 10^−4^ mm^2^/s. This can be explained by the high amount of water and vascularization of hematopoietic marrow accompanied by low amount of fat cells. Normal fatty bone marrow (mean age > 50 years) is characterized by a high signal intensity on T1-weighted images and low signal intensity on fsT2-weighted images due to the high amount of fat cells, and low amount of interstitial water and hematopoietic marrow. This is associated with low signal intensity on b-value images, low ADC values (2.78 × 10^−4^ mm^2^/s) and low perfusion.

In the development of multiple myeloma, there is a gradual increase in monoclonal plasma cells in the bone marrow. The corresponding bone marrow changes are explained in the table by subdividing MM patients in groups of increasing plasma cell percentages: 0–10 %, 10–25 %, 25–50 % and >50 %. Increased plamacytosis in the bone marrow is accompanied by a gradual decrease in fat cells and hematopoietic marrow with an increase in interstitial water. This disease evolution is reflected in the signal intensities: decrease on T1-weighted images together with an increase on fsT2-weighted and b-value images with high ADC values (4.41 × 10^−4^ mm^2^/s, if the plasma cell percentage is higher than 50 %). The increasing tumour load induces neoangiogenesis with corresponding increase in vascularization and perfusion, reflected in the TIC, with a steeper wash in and high amplitude due to the increase in number of vessels and capillary permeability, followed by a wash-out.

Early response to therapy is characterized by oedema and haemorrhage due to cellular death and vascular congestion, inducing a further increase in interstitial water, ADC-values and signal intensity on fsT2-weighted and b-value images, with corresponding decrease in T1 signal intensity. There is a change in TIC with flattening of the wash-in due to a decrease in perfusion, and number of vessels and enhancement during second pass due to the increased interstitial space. Several weeks after therapy, there is a normalization of the bone marrow appearance on MR imaging due to fatty reconversion.

## Pitfalls

A limitation of the qualitative evaluation of DWI is the T2-shine-through effect. The T2-shine-through effect occurs because the measured signal intensity on the high b-value images depends not only on the water proton diffusivity, but also on the intrinsic tissue T2 relaxation time. As a result, a tissue may appear to exhibit high signal intensity on high b-value images, not because of the restricted mobility of the water protons, but because of the long intrinsic T2 relaxation time of the tissue. T2-shine-through is observed in benign conditions such as cysts, post-operative seromas or tissue. Similar appearances can be seen when bone tumours are successfully treated, particularly when there has been massive liquefaction necrosis [27, 51]. In order to avoid misinterpretations arising from visual assessment of the signal intensity on b-value images, it is essential to correlate the findings with the morphological features on the associated conventional MR sequences (Fig 16) [27].

**Fig. 17:**
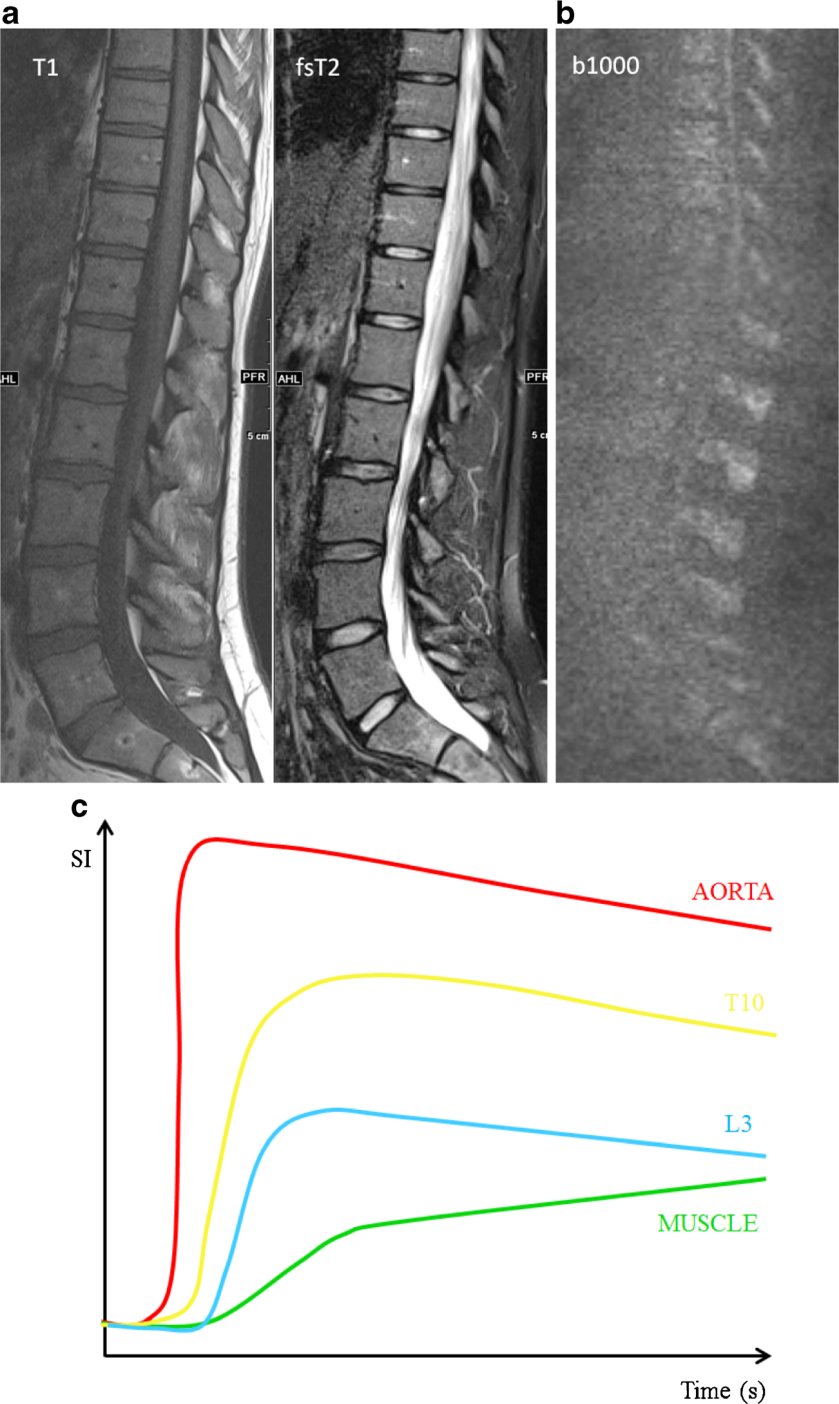
MR images of healthy young subject with normal red bone marrow. **a** The bone marrow appears hypointense on T1-weighted images (*left*), without focal lesions and with a corresponding high signal intensity on fat-suppressed T2-weighted images (*right*). **b** There is a high signal intensity on b1000 images due to the low amount of fat cells, high cellularity (hematopoietic cells) and high amount of water. **c** The vascularization and perfusion are also high (TIC type 4), with an early wash-out of contrast medium

Red bone marrow is characterized by the same signal intensity changes as compared to malignant myeloma infiltrated bone marrow. This presents a potential difficulty in assessing the bone marrow of younger patients and patients receiving bone marrow-stimulating factors after CR [26]. Red bone marrow hyperplasia is also characterized by an increased water diffusivity and vascularization. This is associated with TIC type 3, 4 or 5, mostly type 3, with a less steep wash-in and wash-out due to normal vessel permeability and moderate size of the interstitial space, high signal intensity on b-value images and high ADC-values (Fig 17) [16]. Since the mean age of patients with MM, MGUS or SMM is higher than 50 years, differentiation from normal bone marrow is usually not difficult [16, 20]

Other pathological processes in the bone or bone marrow mimic myeloma lesions by providing the same signal intensity characteristics on conventional MRI images, e.g. bone marrow oedema, subchondral geode, schwannoma, schmorll nodules and scar tissue after bone marrow biopsy. These lesions can be distinguished from myeloma by assessing the localization and other specific features on conventional MRI [27].

## Limitation of MRI

MRI has several disadvantages: relatively high costs and long scanning time, which may be difficult in ill patients [52]. Patients with claustrophobia and metallic implants have to be excluded from this imaging method [8].

A limited examination protocol consists of coronal T1-weighted images of the spine, sagittal T1-weighted and fsT2-weighted or STIR images of the thoracolumbar spine, followed by dynamic contrast-enhanced and diffusion weighted sequences of the thoracolumbar spine, reducing the examination time from 90 to 30 min.

## Conclusion

A complete MRI investigation of bone marrow in patients with plasma cell dyscrasias with conventional whole body MRI, and functional DCE-MRI and DWI sequences of the thoracolumbar spine, provides insights in the composition of the bone marrow. A summarizing table of the imaging methods and bone marrow changes during the course of the disease is provided, including changes in fat cells and water amount, bone marrow cellularity, vascularization, volume of the interstitial space, vessel permeability and water diffusivity. The obtained information on bone marrow changes is of important value in diagnostic work-up and response assessment.

## Abbreviations

^18^F-FDG PET: ^18^F-fluoro-deoxyglucose positron emission tomography
ADC: Apparent diffusion coefficient
DCE: Dynamic contrast-enhanced
DWI: Diffusion weighted imaging
fs: fat-suppressed / fat-saturated
M-protein: Monoclonal protein
MGUS: Monoclonal gammopathy of undetermined significance
MDCT: Multidetector computed tomography
MM: Multiple myeloma
MRI: Magnetic resonance imaging
SE: Spin-echo
SMM: Smouldering multiple myeloma
T1-weighted: T1-weighted spin-echo
T2-weighted: T2-weighted spin-echo
TIC: Time-intensity curve
